# Room Temperature Synthesis of V-Doped TiO_2_ and Its Photocatalytic Activity in the Removal of Caffeine under UV Irradiation

**DOI:** 10.3390/ma12060911

**Published:** 2019-03-19

**Authors:** Olga Sacco, Diana Sannino, Mariantonietta Matarangolo, Vincenzo Vaiano

**Affiliations:** Department of Industrial Engineering, University of Salerno, Via Giovanni Paolo II, 132, 84084 Fisciano (SA), Italy; osacco@unisa.it (O.S.); mariantonietta.mata@gmail.com (M.M.); vvaiano@unisa.it (V.V.)

**Keywords:** V-doped TiO_2_, sol-gel, room temperature synthesis, photocatalysis, water treatment, caffeine

## Abstract

In this work, the influence of simple acids in the room temperature sol-gel synthesis of TiO_2_ was investigated and the efficiency of prepared photocatalysts was evaluated in the removal of caffeine. To improve the photoactivity of TiO_2_, vanadium-doped TiO_2_ (VTiO_2_) samples were obtained starting from different amount of vanadyl sulphate as a dopant source. The samples were centrifuged, washed and finally dried at room temperature, and no calcination step was carried out. The prepared photocatalysts were characterized by different techniques (X-ray powder diffraction (XRD), specific surface area (SSA), ultraviolet-visible diffuse reflectance spectra (UV-vis DRS) and Raman). VTiO_2_ photocatalysts were tested in the photocatalytic removal of aqueous solutions containing caffeine. The photocatalytic tests were carried out in a recirculating batch cylindrical photoreactor irradiated by a UV LEDs strip (nominal power of 12 W and wavelength emission peak at about 365 nm) surrounding the external surface of the reactor. The optimized VTiO_2_ photocatalyst was able to reach a caffeine degradation of about 96% after 360 min of UV light irradiation with a total organic carbon (TOC) removal of 72%.

## 1. Introduction

The design of nanomaterials is a critical issue for industrial applications and their preparation methods largely affect the efficacy of nanotechnology and their application. Among oxide nanomaterials, TiO_2_ is used in a wide range of common and high-tech applications due to its moderate price, chemical stability, non-toxicity, biocompatibility and efficient photocatalytic properties [1]. It was reported that the photocatalytic activity of TiO_2_ depends on crystal size, specific surface area, crystallinity and absorption properties [2]. Generally, among the various crystalline phases of titania, anatase shows a better photocatalytic activity [3]. However, it is well known that anatase TiO_2_, with a small fraction of rutile or brookite phase, showed enhanced photocatalytic activity compared to pure anatase TiO_2_ due to the improved electron and hole separation [4]. In addition, some papers report that anatase-brookite composites were more efficient than anatase-rutile TiO_2_ for the photodegradation of a wide range of organic pollutants [5].

There are several methods for the synthesis of titania and titania based nanomaterials, such as sol-gel method [6], hydrothermal method, chemical vapour deposition (CVD), direct oxidation and microwave methods [7]. Unfortunately, these methods require organic solvents, corrosive chemicals and a high amount of energy to remove the organics used in the preparation of the colloidal suspensions. For this reason, in recent years, efforts have been made to produce titania based nanomaterials through chemical routes, which are less energy consuming and do not require the use of solvents [8].

Several works [9] have been carried out for modifying the sol–gel preparation of crystalline TiO_2_ in acidic aqueous solution at a low temperature (about 50 °C–100 °C), but the photocatalytic activity is generally very low. One method for increasing the activity could be the doping of TiO_2_ structure [10] with metal ions (such as Fe, Cu, Ni, V) [11] that can bring to defects in the semiconductor lattice, increasing the photocatalytic efficiency. A recent paper reports that vanadium doping TiO_2_ provides a potentially promising strategy to improve the properties of photocatalytic materials [1,12]. Caffeine was chosen as model pollutant since it is a psychoactive molecule consumed both for beverages and for pharmaceuticals and personal care products. This pollutant has been detected in natural water in many countries and it is considered an emerging pollutant [13]. Caffeine removal by means of advanced oxidation processes are already shown in the literature [14,15,16]. However, to our knowledge, papers concerning the photocatalytic degradation of this pollutant using photocatalysts prepared at room temperature are still scarce.

For these reasons, in this paper the effects of the vanadium amount in V doped TiO_2_ photocatalysts prepared at room temperature by sol-gel procedure were investigated, and the photocatalytic activity of the samples was studied in the degradation of caffeine under UV irradiation.

## 2. Materials and Methods 

### 2.1. Synthesis and Characterization of TiO_2_ and V-Doped TiO_2_ Photocatalysts

The samples named TiO_2_ AA and TiO_2_ AN were prepared by adding 5 mL of titanium tetraisopropoxide (TTIP) (Sigma-Aldrich S.r.l., Milan, Italy, 98%) dropwise into 100 mL of bi-distilled water containing 10 mL of acetic acid (glacial) or 0.1 mL of nitric acid (Sigma-Aldrich S.r.l., Milan, Italy, 99.8%), respectively. The suspension was vigorously stirred at room temperature (25 °C) for 24 h. Finally, the precipitate was washed with distillate water and then centrifuged. The precipitate was dried at room temperature for 48 h and stored.

The VTiO_2_ samples were obtained starting from different amounts of vanadyl sulphate (0.5, 1 and 5 mg) as a dopant source. In particular, vanadyl sulphate was dispersed in 100 mL of bi-distilled water containing 0.1 mL of nitric acid and then 5 mL of TTIP was added dropwise. The obtained solid phases were decanted and separated, washed with distillate water and then centrifuged for the separation. Finally, the obtained powders were dried at room temperature for 48 h. The nominal content of VTiO_2_ in the final samples was reported in Table 1 in terms of V/Ti molar ratio and crystal phase (A = anatase, B = brookite).

The physical-chemical characterization of the catalysts has been carried out by means of X-ray diffraction analysis (XRD) performed with an X-ray micro-diffractometer Rigaku D-max-RAPID (Rigaku, Tokyo, Japan). Laser Raman spectra were obtained at room temperature with a Dispersive MicroRaman (Invia, Renishaw, Gloucestershire, UK), equipped with a 514 nm laser, in the range 100–800 cm^−1^ Raman shift.

The Brunauer, Emmett and Teller (BET) specific surface area (SSA) of the catalysts was obtained from the dynamic N_2_ adsorption measurement at −196 °C, using a Costech Sorptometer 1042 instrument, after a pre-treatment of the samples at 35 °C for 180 min in He flow. The ultraviolet-visible diffuse reflectance spectra (UV-Vis DRS) were acquired using a Perkin Elmer Lambda 35 spectrophotometer (Perkin Elmer, Waltham, MA, USA) using a RSA-PE-20 reflectance spectroscopy accessory (Labsphere Inc., North Sutton, NH, USA). The band gap values were estimated through the Kubelka-Munk function (KM) (which is proportional to the absorption of radiation) and by plotting (KM × hν)^2^ as a function of hν and evaluating the intercepts on the x-axis of the linear part of the obtained curves.

### 2.2. Photocatalytic Tests

Caffeine (CAF) solutions, at initial concentration equal to 25 mg L^−1^, were prepared by adding 25 mg in 1 L of distilled water.

The photocatalytic tests were performed with a pyrex cylindrical photoreactor (internal diameter, ID = 2.5 cm) equipped with an air distributor device (air flowrate, Q_air_ = 150 cm^3^ min^−1^) and magnetic stirrer to maintain the photocatalyst suspended in the aqueous solution. A UV-LED strip (nominal power 12 W, LED lightinghut) emitting at 365 nm, was used as light source. A volume of CAF solution equal to 100 mL with a catalyst dosage of 3 g L^−1^ was employed for the photocatalytic activity tests.

The LED strip was positioned in contact with the external body of the photoreactor (special glassware on own design realized by Microglass Heim s.r.l., Naples, Italy). Before the irradiation, the suspension was left in dark for 120 min to provide an adsorption/desorption equilibrium on the photocatalyst surface and, after this step, the photocatalytic test began under UV light irradiation up to 360 min.

During the tests, 2 mL of aqueous suspension were withdrawn and centrifuged to remove the catalyst powders to determine the residual CAF concentration at 272 nm by a Perkin Elmer UV-Vis spectrophotometer. The total organic carbon (TOC) contained in a fixed volume of the solution was measured by the high temperature combustion method on a catalyst (Pt-Al_2_O_3_) in a tubular flow micro-reactor which operated at 680 °C. The solution was injected in the catalytic reactor fed with air to oxidize the organic carbon into CO_2_, whose concentration in the gas-phase was monitored by a continuous analyzer (Uras 14, ABB Italia, Milano, Italy) [17].

## 3. Results

### 3.1. Characterization

**Figure 1:**
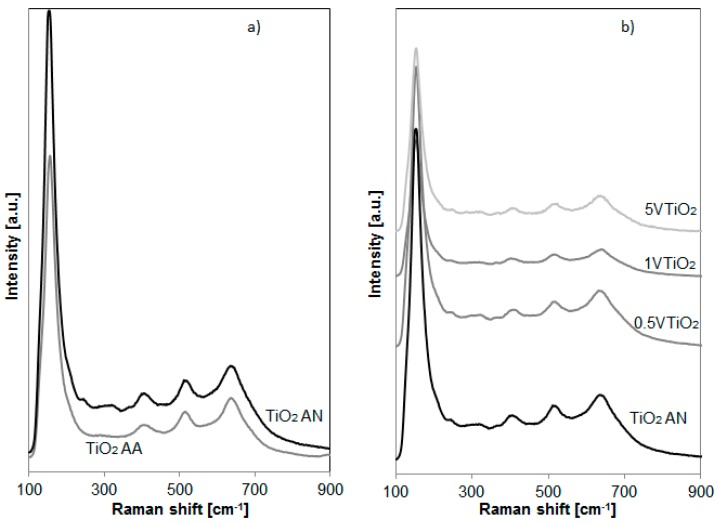
Raman spectra of (**a**) TiO_2_ AA and TiO_2_ AN; (**b**) TiO_2_ AN, 0.5VTiO_2_, 1VTiO_2_ and 5VTiO_2_.

The Raman spectra of the sample are shown in Figure 1. The dominant modes in the Raman spectra of TiO_2_ AA and TiO_2_ AN samples at E_g_ (144 cm^−1^), E_g_ (200 cm^−1^), B_1g_ (397 cm^−1^), B_1g_/A_1g_ (516 cm^−1^), and E_g_ (639 cm^−1^) can be assigned to the Raman active modes of the anatase crystal phase [18]. However, for TiO_2_ AN, it is evident the presence of additional bands at A_1g_ (247 cm^−1^) B_1g_ (320 cm^−1^) that could be attributed to the TiO_2_ in brookite form (Figure 1a) [19].

Figure 1b reports the Raman spectra of VTiO_2_ photocatalysts. It is possible to observe that the presence of vanadium did not induce a change of the TiO_2_ crystalline structure. Moreover the presence of vanadium oxides or other vanadate structures, with bands expected in the range 800–1050 cm^−1^, are not observed [20].

**Figure 2:**
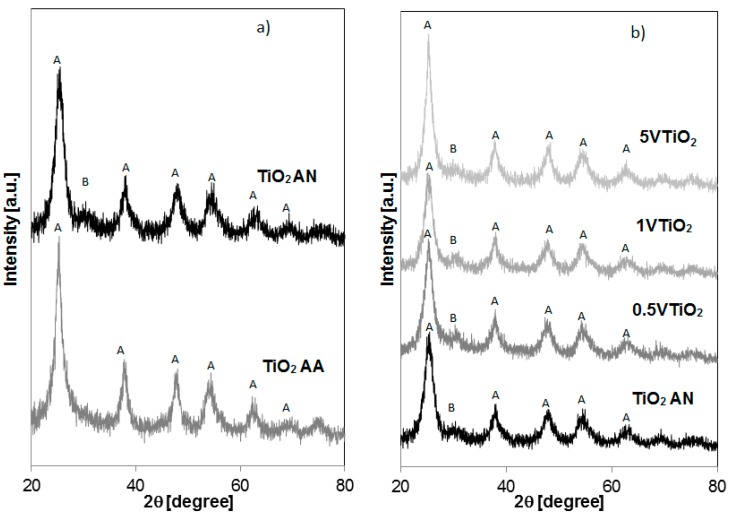
XRD patters of (**a**) TiO_2_ AA and TiO_2_ AN; (**b**) TiO_2_ AN, 0.5VTiO_2_, 1VTiO_2_ and 5VTiO_2_.

The crystalline phases of the samples were also determined by XRD analysis (Figure 2). The TiO_2_ AA sample (Figure 2a) shows diffraction peaks typically of TiO_2_ in anatase form (indicated with capital letter “A” in the Figure 2) at 25.13, 37.6, 47.43, 53.74, 62.06 and 68.06 degrees while TiO_2_ AN shows diffraction peaks due not only to anatase phase but also to brookite (indicated with capital letter “B” in the Figure 2) because of the presence of an additional diffraction signal at 30.8 degree. These results indicate that TiO_2_ AN sample consists of biphasic anatase-brookite nanoparticles [21]. All the peaks found in the case of VTiO_2_ nanoparticles (Figure 2b) are similar to those ones observed for TiO_2_ AN without any additional peaks different from anatase (A) and brookite (B) phases. No diffraction peaks corresponding to V-species is found for all the samples. Additionally, Figure 2b evidenced that the presence of vanadium induced a slight shift of the main peak position of TiO_2_ AN from 25.13 to 25.28 degree (for 1VTiO_2_ sample). The shift of the main TiO_2_ peak to a higher diffraction angle is consistent with the incorporation of V^4+^ ion, whose radius (0.72 Å) is smaller than that of Ti^4+^ ion (0.74 Å) [12]. However, no clear correlation between the V amount and the shift of the main peak of the anatase phase seems evident indicating that V^4+^ ion was incorporated into the crystal lattice of TiO_2_, or vanadium oxides species are very small in size and homogeneously dispersed on the catalyst surface [12]. The TiO_2_ crystallite size and crystalline phase type are listed in Table 1. It can be seen that the TiO_2_ in brookite phase is present only for the sample prepared with nitric acid (TiO_2_ AN) and it is completely absent for TiO_2_ prepared with acetic acid (TiO_2_ AA). The average crystallite size of all the samples was calculated using the Scherrer equation. The calculated crystallite sizes of TiO_2_ AN (5.23 nm) are smaller than TiO_2_ AA (6.93 nm). Typically, the crystal structure and particle sizes depend on different synthesis conditions, such as pH and type of used chemicals [4,8]. Possibly, during the synthesis process, acidic substances could be seen as catalysts able to induce a change in the crystallization mechanism and therefore can influence the final TiO_2_ crystallite size and consequently the photocatalytic activity [22].

**Figure 3:**
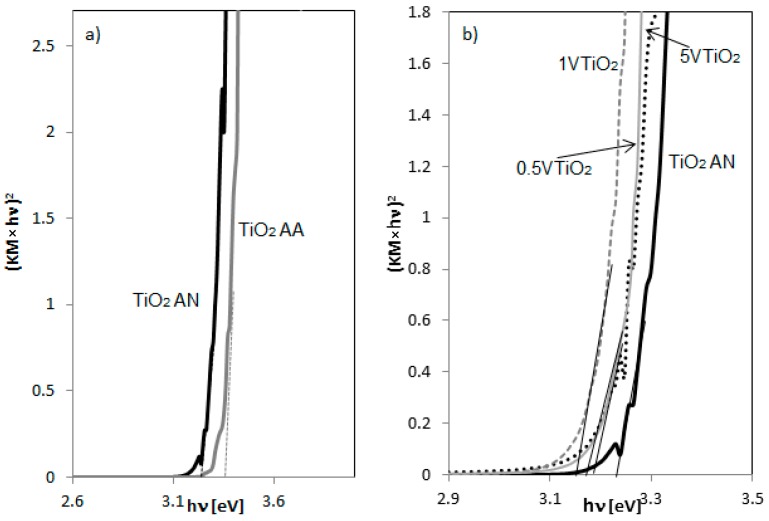
Elaborations of UV–Vis Diffuse Reflectance Spectra for (**a**) TiO_2_ AA and TiO_2_ AN; (**b**) TiO_2_ AN, 0.5VTiO_2_, 1VTiO_2_ and 5VTiO_2_.

The results reported in Table 1 evidenced that the crystallite size for 0.5VTiO_2_ sample (V/Ti molar ratio equal to 1.84 × 10^−4^) is equal to 4.63 nm, lower than the size observed for TiO_2_ AN catalyst (5.23 nm). With the further increase of the V doping level, the crystallite size slightly increased with respect to 0.5VTiO_2_ sample. The specific surface areas (SSA) were also measured for all the samples and reported in Table 1. In particular, the SSA was similar for the sample TiO_2_ AA and TiO_2_ AN and almost equal to about 330 m^2^ g^−1^ while the presence of V in the TiO_2_ crystalline structure led to an slight increase for 0.5VTiO_2_ and a strong decrease for 1VTiO_2_ and 5VTiO_2_ samples. Elaborations of UV-Vis DRS spectra for the evaluation of band gap energy are shown in Figure 3. From this comparison between TiO_2_ AA and TiO_2_ AN (Figure 3a) it can be seen that the addition of nitric acid in the solution synthesis shifted the absorption onset to a lower wavelength compared with the sample prepared using acetic acid, due to the presence of brookite phase in TiO_2_ AN sample [23]. Figure 3b reports the elaborations of UV-Vis DRS spectra for VTiO_2_ samples, evidencing that the introduction of V^4+^ ion in the TiO_2_ structure led to a decrease of the absorption band edge compared to TiO_2_ AN.

The band gap values, estimated from the diffuse-reflectance spectra using the Kubelka-Munk function, are reported in Table 1. A small decrease is observed after the doping with vanadium. The decrease of band gap values observed for V-doped TiO_2_ samples is due to the charge-transfer transition between the d electrons of the vanadium dopant and the conduction band (or valence band) of the TiO_2_ [12].

### 3.2. Photocatalytic Activity Tests

**Figure 4:**
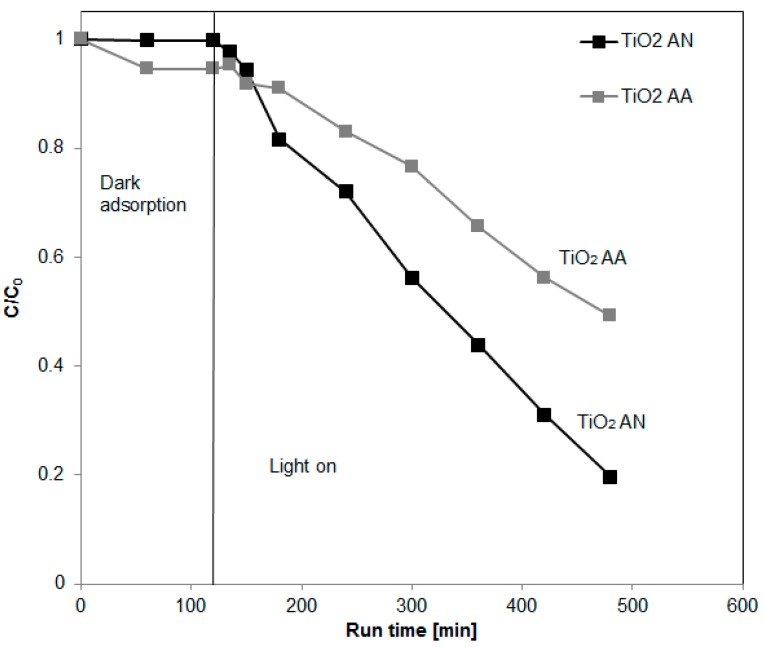
Photocatalytic degradation of caffeine using TiO_2_ AA and TiO_2_ AN under UV irradiation.

Figure 4 shows the photocatalytic results of CAF degradation using TiO_2_ AA and TiO_2_ AN photocatalysts under UV irradiation.

**Figure 5:**
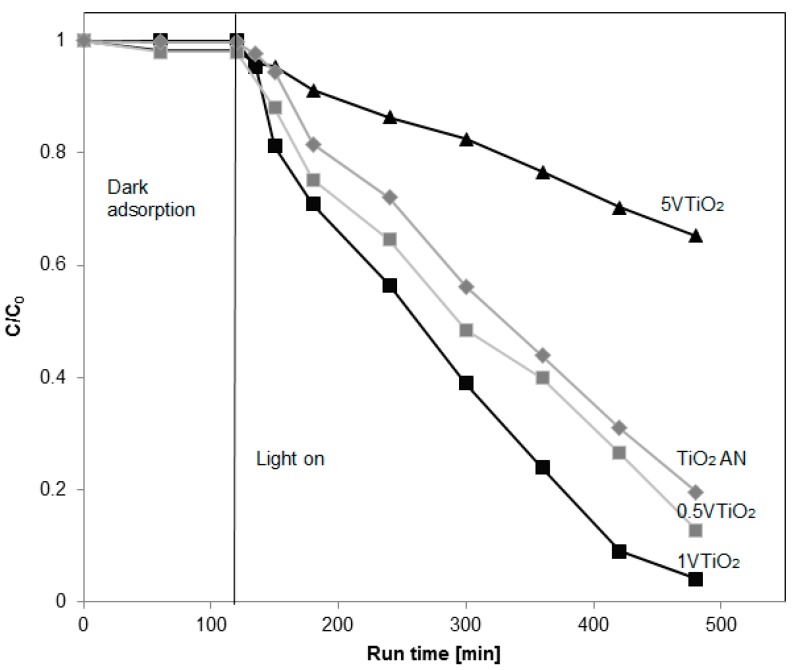
Photocatalytic degradation of caffeine using TiO_2_ AA and doped VTiO_2_ (0.5VTiO_2_, 1VTiO_2_ and 5VTiO_2_) under UV irradiation.

In particular, the best results were obtained using TiO_2_ AN sample leading to a degradation of 80% after 360 min of UV irradiation. A lower activity was achieved for TiO_2_ AA photocatalyst (caffeine degradation of 51% after 360 min of UV irradiation). This result could be due to the presence of biphase (anatase, brookite) that allowed a slight decrease in the TiO_2_ AN particle size and decrease in the band-gap energy (Table 1). The coupling of different TiO_2_ crystalline phases allows the displacement of electrons from one semiconductor to another, leading to more efficient electron/hole separation and enhancing the photocatalytic reactivity [24]. The effect of V content was also analyzed in terms of CAF degradation (Figure 5). It can be seen that, after 360 min of UV irradiation, the photocatalytic CAF degradation increased from 87% to 96% by increasing the V content (sample 0.5VTiO_2_ and 1VTiO_2_ samples), showing photocatalytic activity higher than that of the undoped TiO_2_ AN (81% of CAF degradation) after the same irradiation time. For a further increase of V content (5VTiO_2_ sample), the photocatalytic activity dramatically decreased, reaching 35% of CAF degradation after 360 min of UV irradiation. In summary, the results with the V-doped TiO_2_ samples showed an optimum doping level able to assure the best activity (96% of CAF degradation after 360 min).

#### Kinetics Evaluation of Pollutants Degradation and Mineralization

In order to assess the influence of the doping level on photocatalytic performances, the kinetic constant of caffeine degradation was calculated (Table 2). It was considered that the CAF photodegradation process can be described by the pseudo-first order kinetics [25]. The photodegradation rate (r) depends on the initial pollutant concentration (C) in accordance with the following equation (Equation (1)):

r = k × C(1)

where C is the concentration of CAF during the UV light in mg·L^−1^ and k is the kinetic constant in min^−1^. Considering the mass balance for the batch reactor (Equation (2)):

(2) $$\frac{\mathrm{dC}}{\mathrm{dt}}=-k\times C$$

and integrating the Equation (2) between initial time (t = 0) and a generic irradiation time t, it was obtained the following equation (Equation (3)):

(3) $$-\ln(\frac{C}{C_{0}})=k\times t$$

The value of the kinetic constant k can be calculated by the slope of the straight line obtained from plotting −ln ($\frac{C}{C_{0}}$) versus irradiation time (t). The obtained values of k for all the investigated photocatalysts are reported in Table 2 (R^2^ values are in the range 0.96–0.99). As can be seen in Table 2, the highest value of k (0.0075 min^−1^) was obtained using the 1VTiO_2_ photocatalyst, evidencing the existence of an optimal V doping content. Moreover, the kinetic constant increased from 0.0039 min^−1^ for TiO_2_ AN to 0.0075 min^−1^ for 1VTiO_2_, while the further increase in V amount (5VTiO_2_ sample), led to a decrease of k value up to 0.0011 min^−1^. Generally, the variation of photocatalytic activity with the increase of dopant content can be ascribed to the cooperative effect between the band gap, crystallite sizes, and crystallinity [12]. However, in this study, the optimal 1VTiO_2_ photocatalyst presents a specific surface area and crystallite size lower than 0.5VTiO_2_ sample and a band gap value lower than TiO_2_ AN (Table 1). Therefore, the presence of an optimum value for V content could be explained considering that the V doping could probably reduce the electron-hole recombination of and thus improves both the CAF photocatalytic degradation and mineralization rates [12]. Moreover, the presence of V in the TiO_2_ lattice can produce more photoinduced electrons and holes, which can increase the photocatalytic activity to some extent [12]. This happened when the V content was increased up to 3.67 × 10^−4^ V/Ti molar ratio. Meanwhile, when the doping level was further increased, the presence of the dopant may induce the formation of recombination centers. Consequently, the recombination of the photogenerated electron-hole pairs could become easier and worsening the photocatalytic activity [26]. Based on the calculated k values from Equation (2), the half-life time (t_1/2_, min) of the CAF photodegradation was determined according to the Equation (4) [27]:

(4) $$t_{1/2}=\frac{\ln2}{k}$$

From the obtained results (Table 2) it can be deduced the half-life time value is sensibly lower for the sample 1VTiO_2_. In addition, in order, to characterize the mineralization ability of the tested samples, TOC measurements were carried out. Table 2 reports the TOC removal obtained after 360 min of UV irradiation. Similar to the degradation results, the best results in terms of TOC removal was obtained using 1VTiO_2_, allowing to reach 72% TOC removal and evidencing, therefore, the ability of the optimized 1VTiO_2_ photocatalyst in the mineralization of the target pollutant.

## 4. Conclusions

In this work, TiO_2_ based photocatalysts were obtained at room temperature starting from a modified sol-gel method. The characterization data showed that the presence of nitric acid during the synthesis induced the formation of a biphase crystalline structure (anatase-brookite) TiO_2_. The best results using bare titania in the photocatalytic removal of caffeine were obtained on the biphase TiO_2_ AN sample, with a degradation of 80% after 360 min of UV irradiation. Vanadyl sulphate was used as a dopant source for increasing the activity of biphase TiO_2_. The XRD data showed that vanadium was incorporated in the crystalline structure of TiO_2_. The presence of vanadium into the TiO_2_ structure significantly enhanced the photocatalytic performances, allowing the achievement of a caffeine degradation of 96% after 360 min of UV irradiation on 1VTiO_2_. The coupling of different TiO_2_ crystalline phases and doping with vanadium induces a lower band gap energy value and permits a more efficient electron/hole separation, enhancing the photocatalytic reactivity. The sample 1VTiO_2_ possesses the optimal trade-off among band gap energy, specific surface area and crystallinity.

## Figures and Tables

**Table 1:** Summary of physicochemical properties of TiO_2_ and VTiO_2_ samples.

No.	V/Ti Molar Ratio	Crystal Phase	Crystallite Size, [nm]	Specific Surface Area, [m^2^/g]	Band Gap Energy, [eV]
TiO_2_ AA	-	A	6.93	326	3.3
TiO_2_ AN	-	A/B	5.23	333	3.2
0.5VTiO_2_	1.84 × 10^−4^	A/B	4.63	350	3.1
1VTiO_2_	3.67 × 10^−4^	A/B	5.21	219	3.1
5VTiO_2_	1.84 × 10^−3^	A/B	5.98	209	3.1

**Table 2:** Kinetic constant (k) and half-life time (t_1/2_) values for degradation process with together TOC removal after 360 min of UV irradiation.

Substance	Catalysts	Degradation		TOC ^1^, %
		k, min^−1^	t_1/2_, min	
CAF	TiO_2_ AA	0.0016	433	9
	TiO_2_ AN	0.0039	177	17
	0.5VTiO_2_	0.0047	147	54
	1VTiO_2_	0.0075	92	72
	5VTiO_2_	0.0011	693	4

^1^ TOC removal after 360 min of UV light irradiation.
